# Harnessing Rare Actinomycete Interactions and Intrinsic Antimicrobial Resistance Enables Discovery of an Unusual Metabolic Inhibitor

**DOI:** 10.1128/mbio.00393-22

**Published:** 2022-05-24

**Authors:** Dylan J. McClung, Yongle Du, Dominic J. Antonich, Bailey Bonet, Wenjun Zhang, Matthew F. Traxler

**Affiliations:** a Department of Plant and Microbial Biology, University of California, Berkeley, California, USA; b Department of Chemical and Biomolecular Engineering, University of California, Berkeley, California, USA; Baylor College of Medicine

**Keywords:** actinomycetes, antimicrobial activity, computational enzyme modeling, microbial interactions, natural products, specialized metabolism

## Abstract

Bacterial natural products have historically been a deep source of new medicines, but their slowed discovery in recent decades has put a premium on developing strategies that enhance the likelihood of capturing novel compounds. Here, we used a straightforward approach that capitalizes on the interactive ecology of “rare” actinomycetes. Specifically, we screened for interactions that triggered the production of antimicrobials that inhibited the growth of a bacterial strain with exceptionally diverse natural antimicrobial resistance. This strategy led to the discovery of a family of antimicrobials we term the dynaplanins. Heterologous expression enabled identification of the dynaplanin biosynthetic gene cluster, which was missed by typical algorithms for natural product gene cluster detection. Genome sequencing of partially resistant mutants revealed a 2-oxo acid dehydrogenase E2 subunit as the likely molecular target of the dynaplanins, and this finding was supported by computational modeling of the dynaplanin scaffold within the active site of this enzyme. Thus, this simple strategy, which leverages microbial interactions and natural antibiotic resistance, can enable discovery of molecules with unique antimicrobial activity. In addition, these results indicate that primary metabolism may be a direct target for inhibition via chemical interference in competitive microbial interactions.

## INTRODUCTION

Despite being interrogated for decades, microbes remain one of the most prolific sources of useful natural products (also called specialized metabolites). A key realization of the postgenomic era has been that many bacterial genomes, including those of actinomycete bacteria, encode a wide array of natural product biosynthetic gene clusters for which the chemical products have yet to be identified (1). These “silent” gene clusters are largely thought to be poorly expressed in axenic cultures under laboratory conditions (2–4). Thus, designing approaches that enhance the likelihood of capturing novel natural products made by these organisms/gene clusters is an area of strong interest.

The past decade has seen the development of multiple new strategies aimed at accelerating natural products discovery (5). Key among these strategies have been genome mining tools that enable targeted studies of gene clusters that are likely to produce novel compounds (3, 6–8). While this approach has proven effective in many example cases, one caveat to such efforts is the reliance on *a priori* gene cluster identification, which may miss atypical gene clusters that encode unique chemical scaffolds. Other studies have capitalized on the idea that silent gene clusters may require ecological cues for their stimulation and subsequent production of their respective specialized metabolites (4, 9, 10). Such efforts have included housing microbes in devices that allow their individual cultivation *in situ* (e.g., via iChips) (11–13), prospecting for unique molecules in the context of chemical symbioses (14–20), the use of microbial cocultures (2, 21, 22), and harnessing compound libraries as elicitors of silent gene cluster activation (HiTES) (23, 24).

The most commonly isolated actinomycetes from soil are the members of the genus *Streptomyces*. In the “golden age” of antibiotics discovery, *Streptomyces* species were deeply sampled, leading to the discovery of a wealth of medically useful natural products (25). However, within the last decade, interactions between *Streptomyces* isolates have been shown to stimulate production of novel compounds, suggesting that interactions among actinomycetes can be a path toward activating silent biosynthetic gene clusters (reviewed in references 26, 27, and 28). The so-called “rare” actinomycetes, including many members of the family *Micromonosporaceae*, have been comparatively underexplored due to their apparent lower frequency in soil and lower growth rates relative to *Streptomyces* spp. (10, 29, 30). Multiple genera of *Micromonosporaceae*, including *Actinoplanes*, *Couchioplanes*, *Dactylosporangium*, and *Kineosporia*, produce flagellated spores, which enable targeted enrichment followed by cultivation of these taxa (31, 32). Taxa within this family are responsible for the production of the medically relevant antimicrobials teicoplanin (33) and ramoplanin (34), and the antidiabetic compound acarbose (35), among others (30). These unusual organisms may represent fruitful sources for natural products discovery, both through continued genomic exploration and via studies of interactions between them.

In natural settings such as soil, microbes live in interactive communities where competition for resources may be strong, and interference competition via secreted molecules appears to be common (36–39). One result of this competitive landscape is the high frequency of antimicrobial resistance genes in the soil metagenome (40, 41), including in the genomes of actinomycetes (42, 43). In such an environment, organisms are likely under pressure to diversify the types of molecules they produce, and the molecular targets exploited by these compounds (10, 16, 44). One way of increasing the probability that natural products discovery efforts return novel compounds may be to screen against organisms that are naturally resistant to multiple antimicrobials. For example, compounds that are effective at inhibiting the growth of naturally resistant competitors may stand a higher chance of having unusual mechanisms of action.

Here, we combined several aspects of actinomycete ecology into a straightforward discovery approach, with the aim of increasing the likelihood of finding novel compounds. This strategy included screening for molecules produced during interactions among rare actinomycetes. Specifically, we screened for induced production of molecules that inhibited the growth of a target actinomycete strain that is resistant to a large number of antibiotics. A small screen piloting this approach yielded a strain of Couchioplanes caeruleus that produced a family of antimicrobials we term the dynaplanins. Dynaplanins appear to target the E2 subunit of a 2-oxo acid dehydrogenase in a subset of actinobacteria, namely, other actinomycetes. Taken together, our results show that leveraging multiple aspects of actinomycete ecology in a single, simple discovery pipeline can yield novel molecules with unusual mechanisms of action. These results also suggest that chemical inhibition of primary metabolic pathways may represent a strategy for interference competition during microbial interactions.

## RESULTS

### A small-scale screen identifies *Couchioplanes caeruleus* as a producer of a narrow-spectrum antimicrobial.

To begin a pilot for a larger screening effort, we selected eight actinomycetes representing broad phylogenetic diversity, with an emphasis on rare actinomycetes, from a collection of soil isolates. Sequencing of 16s rRNA genes tentatively identified these organisms as Couchioplanes caeruleus (strain T141), *Actinoplanes* sp. (strain T137), *Micromonospora* sp. (strain T104), *Blastococcus* sp. (strain T139), *Cryptosporangium* sp. (strain T115), *Kineococcus* sp. (strain T115), *Microlunatus* sp. (strain T131), and *Streptomyces* sp. (strain T091) (see Table S1 in the supplemental material at https://doi.org/10.6084/m9.figshare.19252106.v1). These strains were predominantly isolated using a strategy designed to enrich for actinomycetes that produce motile spores (32).

**FIG 1:**
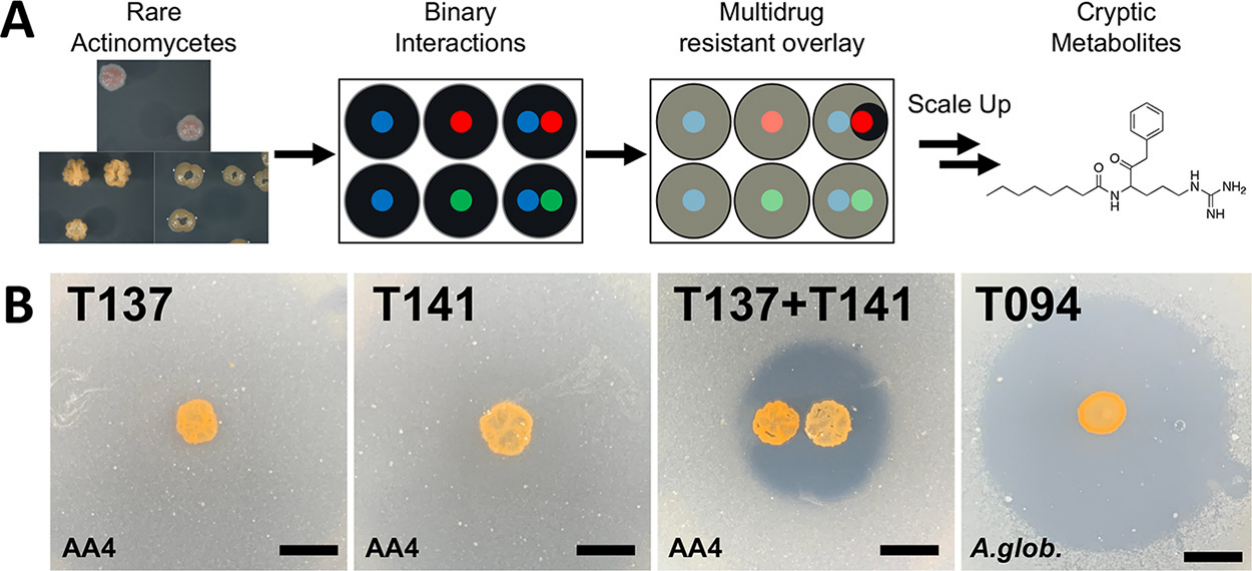
Couchioplanes caeruleus soil isolates produce an unknown antibacterial, dynaplanin. (A) Overview of workflow for the discovery of dynaplanins. Eight wild soil isolates of mostly rare actinomycetes (see strain details in Table S1) were placed in binary interactions and overlaid with a multidrug-resistant bacterium, *Amycolatopsis* sp. AA4. Of the 28 unique interactions, only one produced an interaction-induced antibiotic, which we further studied to identify the dynaplanins. (B) A patch of Actinoplanes sp. isolate T137 alone, and a patch of *C. caeruleus* isolate T141 alone do not produce detectable antibacterial molecules against *Amycolatopsis* sp. AA4 when grown on ISP2. T137 stimulates T141 to produce an unknown antibacterial against AA4. Another *C. caeruleus* isolate (T094) produces this antibacterial alone against *Arthrobacter globiformis* when grown on ISP4g+MOPS. Bacterial species in the soft agar overlay is indicated in the bottom left corner. Scale bar, 5 mm. Other *Couchioplanes* strains and EICs of dynaplanin A (molecule 1) are shown in Fig. S1.

For screening, the strains were grown on solid ISP2 medium as patches in multiwell plates. Each strain was grown alone and in binary interactions with every other strain, totaling 28 unique interactions (Fig. 1A). After 5 days of growth, the patches were overlaid with soft agar containing an indicator strain to check for antibiotic production, as originally described by Ueda et al. (45). For the indicator strain, we used *Amycolatopsis* sp. strain AA4, which was originally identified based on its extraordinary multidrug resistance profile, which includes resistances to at least 15 different antibiotics (42). After incubation overnight with the *Amycolatopsis* sp. AA4 overlay, a visible halo of antibiotic production was observed in a single interaction in which *Actinoplanes* sp. strain T137 stimulated Couchioplanes caeruleus T141 to produce an antimicrobial (Fig. 1B). Notably, neither of these organisms produced an antimicrobial when grown alone as individual patches.

We sought to optimize production and detection of this antimicrobial for chemical purification. We found that other Couchioplanes caeruleus isolates collected from different locations (T094 and T095) and the type strain DSM43634 also produced antimicrobial activity against *Amycolatopsis* sp. AA4, even when grown alone. The antimicrobial produced by these strains showed activity against a range of actinobacterial target strains but did not inhibit other Gram-positive or negative bacteria or fungal strains (Table 1; see also Table S2). Among the actinobacteria inhibited by this antimicrobial, Arthrobacter globiformis (ATCC 8010) exhibited strong sensitivity (Fig. 1B; see also Fig. S1A) and a relatively high growth rate, making it an ideal test organism for use in assay-guided fractionation/purification.

**TABLE 1:** MIC of dynaplanin A

Species	MIC^*a*^	
	μg/mL	μM
Arthrobacter globiformis	200	534.4
*Amycolatopsis* sp. AA4	50	133.6
Escherichia coli	>200	>534.4
Bacillus subtilis	>200	>534.4

a MICs were determined by the wells with the lowest concentration of dynaplanin A that resulted in no growth of the species compared to a dynaplanin A blank control.

### Structural elucidation of the dynaplanins.

**FIG 2:**
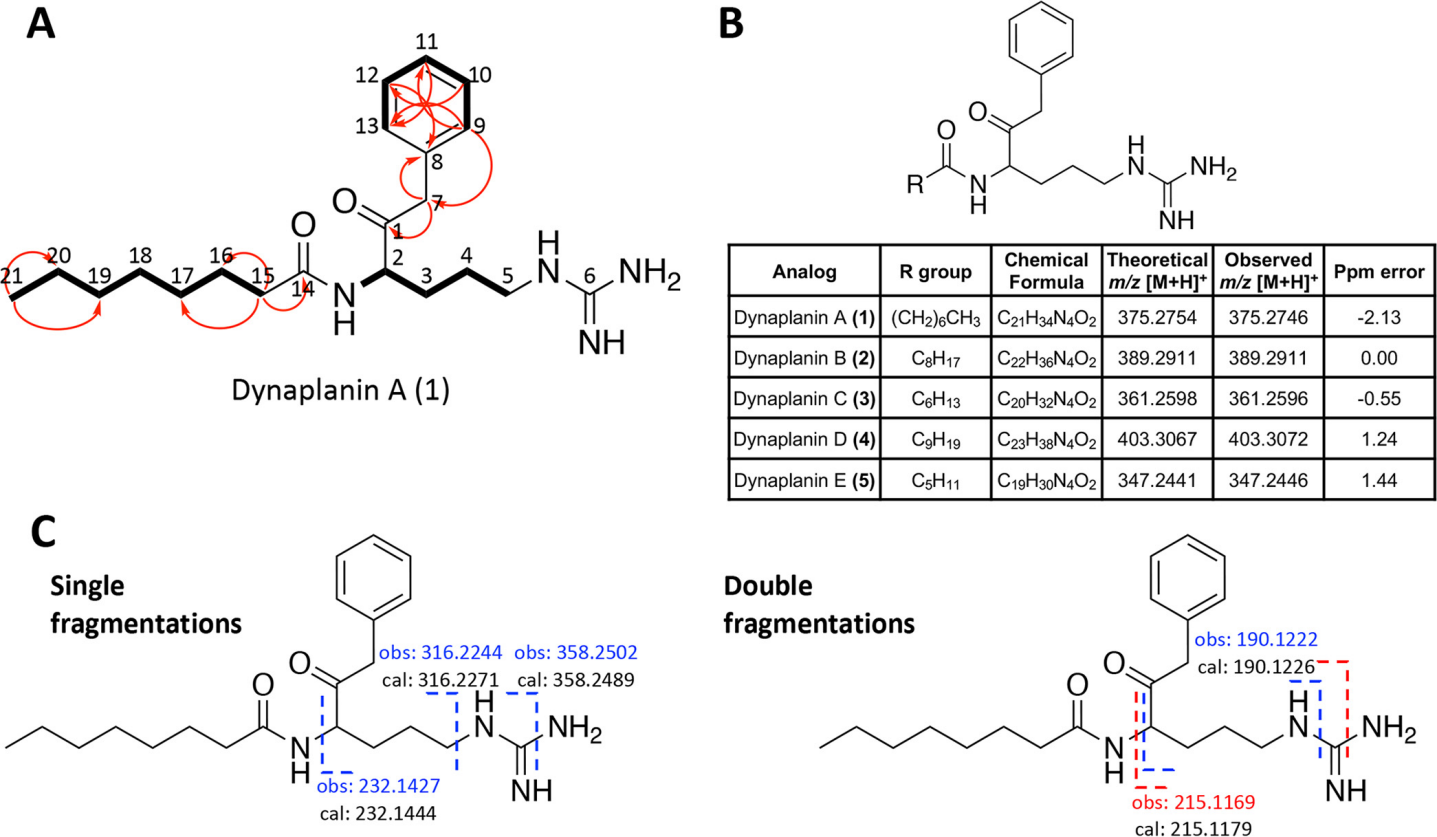
Structural elucidation of dynaplanins A to E. (A) Key COSY (thick bonds) and HMBC (red arrows) correlations from dynaplanin A experiments. (B) Structure of dynaplanin core and details for each analog. Theoretical *m/z* of [M+H]^+^ values for all analogs and ppm errors were calculated by the Barrows group online calculator tool. Chemical formulas represent neutral species. (C) MS2 fragmentation of dynaplanin A with observed diagnostic peaks (obs, top) and theoretically calculated (cal, bottom). Only [M+H]^+^ ions from single (left) and double (right) bond fragmentations are shown. An MS2 spectrum is shown in Fig. S2A, and further fragmentation is explained in Table S4.

High-resolution mass spectrometry (HR-MS) analysis of ethyl acetate extracts showed that a candidate feature with an *m/z* of 375.2746, corresponding to the molecular formula C_21_H_34_N_4_O_2_, was present in all *C. caeruleus* cultures (i.e., *C. caeruleus* T141 interacting with *Actinoplanes* sp. T137, and *C. caeruleus* strains T094, T095, and DSM43634 grown in isolation; Fig. S1B). Assay-guided fractionation via preparative-scale HPLC led to further purification of 0.5 mg of this compound, *N*-(6-guanidino-2-oxo-1-phenylhexan-3-yl)octanamide, which we named dynaplanin A (molecule 1), from 3.5 L of solid cultures of *C. caeruleus* T094, enabling characterization via nuclear magnetic resonance spectroscopy (Fig. 2A; see also Table S3). Dynaplanin A ^1^H NMR and HSQC spectra displayed signals for one arginine moiety (d_H_ 8.51, -CO-NH-; d_H_ 4.28, H-2; d_H_ 3.05, H-5a; d_H_ 3.01, H-5b; d_H_ 1.71, H-3a; d_H_ 1.54, H-3b; d_H_ 1.49, H-4), one benzyl group (d_H_ 7.28, H-10 and H-12; d_H_ 7.21, H-11; d_H_ 7.12, H-9 and H-3; d_H_ 3.81, H-7a; d_H_ 3.78, H-7b), and one linear long chain (d_H_ 7.28, H-15; d_H_ 1.50, H-16; d_H_ 1.20 to 1.25, H-17, H-18, H-19, and H-20; d_H_ 0.83, H-21). The benzyl group was connected to C-1 based on ^2^*J* HMBC cross-peak from H-7a/b to C-1 (d_C_ 207.1); meanwhile, the long chain was linked to C-14, as indicated by ^2^*J* HMBC correlation between H-15 and C-14 (d_C_ 172.8). Finally, the configuration of C-2 remains unassigned. The structure determined by nuclear magnetic resonance (NMR) was further corroborated by HR-MS/MS, which showed strong correlations between mass-to-charge ratios for observed and predicted fragment ions (Fig. 2C; see also Table S4). HR-MS/MS also enabled the identification of several related analogs, which varied only in the composition of the acyl chain. These analogs were named dynaplanin B-E according to their relative abundance (Fig. 2B). Nine additional analogs possessed acyl-chain modifications that we speculate to include variable degrees of unsaturation, possible ring formation, and oxidations (i.e., hydroxyl, ether, or ester moieties; Table S5). The molecular formulas for the nine additional analogs were validated by exact mass within 5 ppm (see Table S5).

To further validate the chemical structure of dynaplanin A and gain insights into dynaplanin biosynthesis, we undertook a series of isotopic labeling experiments. For these experiments, *C. caeruleus* T094 was grown on solid modified ISP4 medium supplemented with l-[^13^C_6_]-arginine, l-[^13^C_9_^15^N]-phenylalanine, or [1,2,3,4-^13^C_4_]-octanoic acid. In all supplementation conditions, HR-MS/MS analysis of crude extracts showed incorporation of stable isotopes into dynaplanin A (see Fig. S2), confirming arginine, phenylalanine, and octanoic acid as precursors in dynaplanin biosynthesis. Dynaplanins B to E also showed incorporation of arginine and phenylalanine, but none of the analogs incorporated octanoic acid (see Fig. S3), as expected given that these analogs differ in the composition of their acyl moieties. In addition, the pattern of incorporation unambiguously showed that C-1 originates from the α-carbon of phenylalanine, indicating that the carbonyl carbon of arginine is removed through decarboxylation, during dynaplanin biosynthesis.

### Identification of the dynaplanin biosynthetic gene cluster.

To identify the gene cluster responsible for dynaplanin biosynthesis, we sequenced the genome of *C. caeruleus* T094 and analyzed it using typical algorithms for finding natural product biosynthesis genes (e.g., antiSMASH). This genome analysis did not identify any gene clusters that seemed relevant to dynaplanin biosynthesis. Specifically, no clusters encoded putative enzymes that were likely to incorporate arginine into the chemical product.

**FIG 3:**
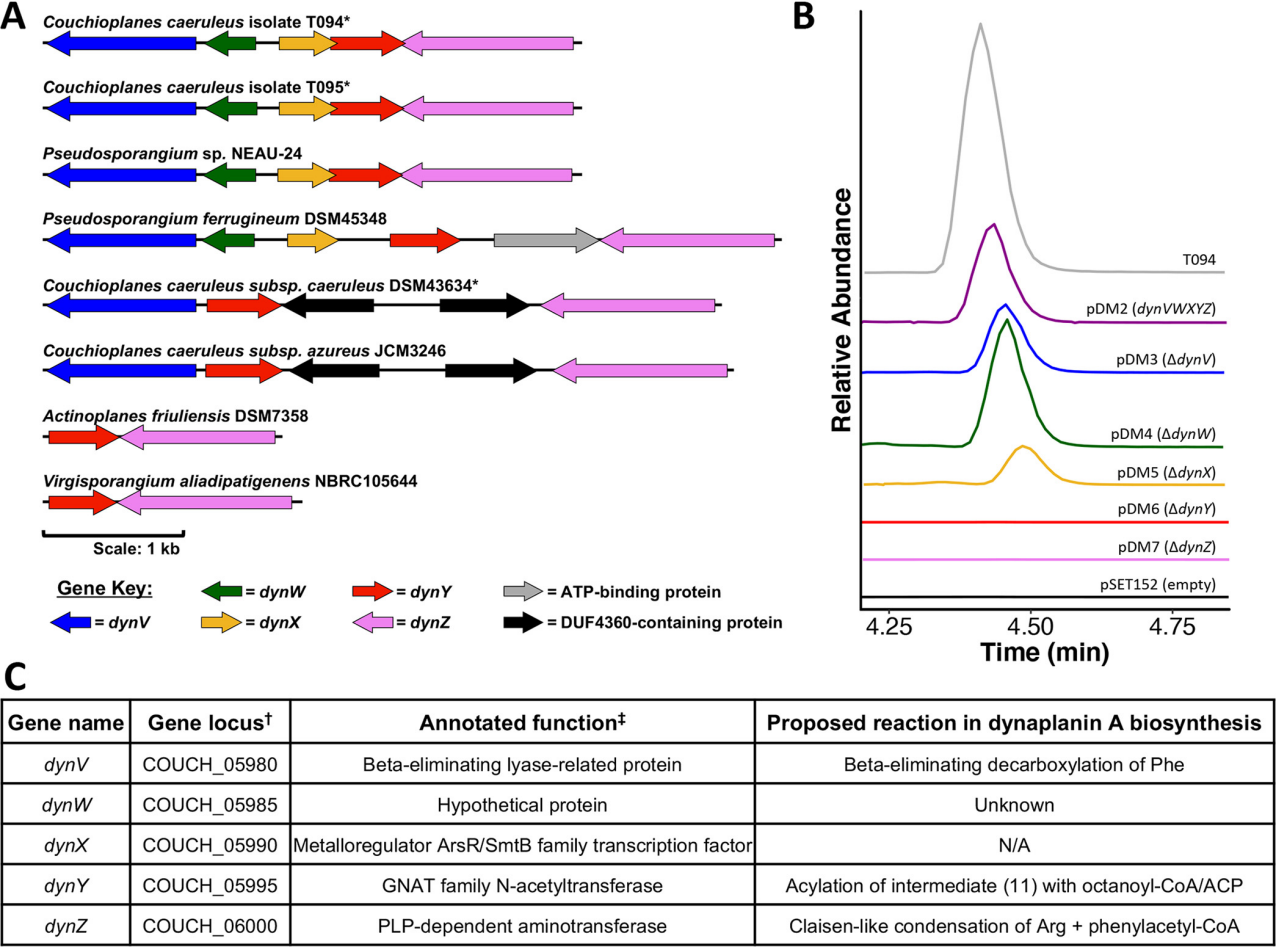
Dynaplanins are produced from a five gene cluster *dynVWXYZ*. (A) The gene organization of *dynVWXYZ* in *C. caeruleus* T094 (top) and a variety of other rare actinomycetes. Gene locus tags: T094, see part C; T095, NA, genes sequenced by PCR/Sanger; NEAU-24, IT780_RS35960–RS35940; DSM45348, CLV70_RS09715–RS09690; DSM43634, EDD30_RS24695–RS24715; JCM3246, IEY99_RS02025–RS02005; DSM7358, AFR_RS17140–RS17135; NBRC105644, Val02_RS09255–RS09250. *, Dynaplanin A production confirmed by LC-MS/MS. Scale bar, 1 kb. (B) EICs of dynaplanin A (375.2746 ± 5 ppm) from S. coelicolor M145 heterologous hosts grown on ISP4g+MOPS for 5 days, except M145+pDM5 which was grown on ISP2 (additional ISP2 controls shown in Fig. S4). The relative abundances of all EICs were normalized to T094 (gray). (C) Gene table of the *dynVWXYZ* cluster. †, Gene locus from T094 genome; ‡, annotations determined by the NCBI Prokaryotic Genome Annotation Pipeline (PGAP).

Given the pattern of isotope incorporation observed, we hypothesized that the C-C bond between C-1 and C-2 was likely the result of a Claisen-type condensation reaction catalyzed by a pyridoxal 5′-phosphate (PLP)-dependent *C*-acetyltransferase. While several PLP-dependent enzymes are known to be involved in natural product biosynthetic pathways (46), one enzyme that catalyzes a reaction similar to the hypothesized reaction is KtmB, which is critical in the biosynthesis of the ketomemicin pseudotripeptides (47). We next searched the genome of *C. caeruleus* T094 for KtmB homologs. This search revealed a candidate PLP-dependent *C*-acetyltransferase whose gene (COUCH_06000, here named *dynZ*) was located next to a gene encoding a GNAT family *N*-acetyltransferase (COUCH_05995, here named *dynY*) (Fig. 3A). We noted that DynZ appears to have strongly conserved homologs across multiple taxa of rare actinomycetes. Comparison of the surrounding genes in several rare actinomycete genomes, including the available genome of *Pseudosporangium* sp. NEAU-24, and a sequence generated via PCR from *C. caeruleus* T095, showed a conserved 5-gene cluster that included the PLP-dependent *C*-acetyltransferase and GNAT family *N*-acetyltransferase (Fig. 3A).

To investigate the role of these genes, which we named *dynVWXYZ*, we cloned and heterologously expressed them in Streptomyces coelicolor M145, which is not a native producer of dynaplanins. HR-MS/MS analysis of extracts from the S. coelicolor strain carrying the five-gene *dyn* cluster via an integrative plasmid (pDM2) confirmed that it produced detectable amounts of dynaplanin A (Fig. 3B), while the strain carrying the empty vector did not. These results indicate that genes within the *dyn* locus are sufficient for dynaplanin production in a heterologous host. We next sought to determine which of the *dyn* genes were required for dynaplanin production. To answer this question, we made five variants of pDM2, each of which had one *dyn* gene deleted, and introduced them into the S. coelicolor host. Analysis of extracts from these strains showed that *dynV, dynW*, and *dynX* were not essential for dynaplanin A biosynthesis, whereas *dynY* and *dynZ* were both required (Fig. 3B). Note the elution time of dynaplanin A from the Δ*dynX* mutant is shifted due to growth on ISP2 (Fig. 3B), since this strain was not viable on ISP4g+MOPS medium. A control from S. coelicolor + pDM2 grown on ISP2 confirms this delay (see Fig. S4).

Further analysis of the *dyn* gene cluster in other actinomycetes showed variability in this locus. *dynV*, *dynW*, and *dynX* were initially annotated to encode a beta-eliminating lyase-related protein, a hypothetical gene of unknown function, and a transcriptional regulator, respectively (Fig. 3C). Notably, two Couchioplanes caeruleus genomes (DSM43634 and JCM3246) lacked *dynW* and *dynX*, while genomes of *Actinoplanes friulensis* and Virgisporangium aliadipatigenens contained minimal clusters consisting of only *dynY* and *dynZ* (Fig. 3A). This pattern of variability, wherein *dynY* and *dynZ* are the only genes conserved across all versions of this locus, is consistent with their essentiality and sufficiency for dynaplanin production.

### A proposed pathway for dynaplanin production.

**FIG 4:**
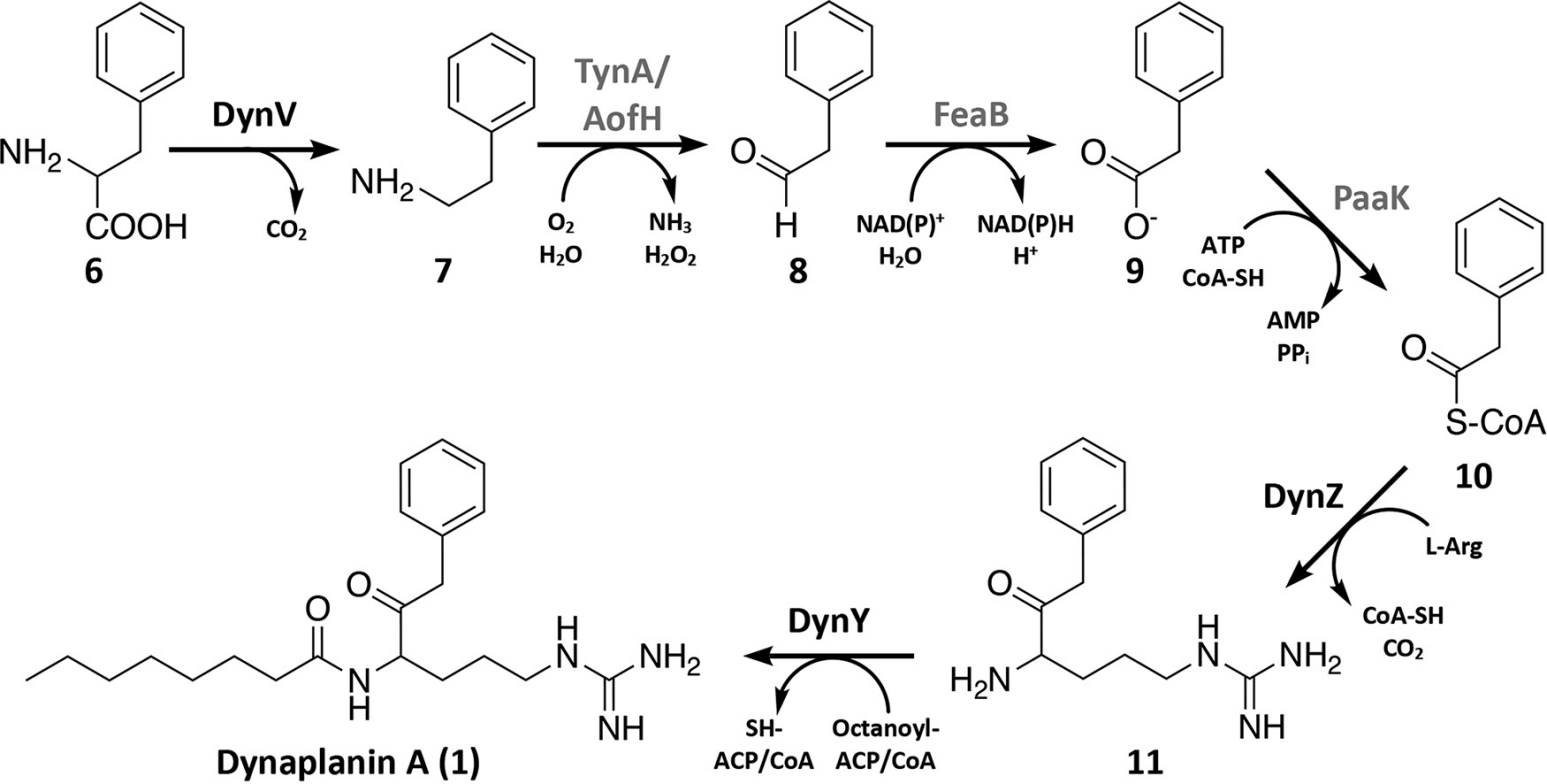
Model of dynaplanin A biosynthesis. We predict that phenylalanine (molecule 6) is decarboxylated by DynV to phenylethylamine (molecule 7) where it is further processed by genes outside the *dynVWXYZ* cluster (gray text; gene details in Table S7). Phenylacetyl-CoA (molecule 10) and l-arginine are bonded by DynZ in a Claisen-like condensation reaction that eliminates the carboxylic acid of arginine to produce an intermediate, 1-(4-amino-5-oxo-6-phenylhexyl)guanidine (molecule 11). DynY performs a condensation reaction to bind octanoyl-ACP/CoA to the amine of the arginyl group to produce dynaplanin A (molecule 1). In dynaplanin B to E biosynthesis, we predict that octanoyl-ACP/CoA is replaced by a fatty acyl-ACP/CoA of variable chain length.

Given the predicted functions of the enzymes encoded by the *dyn* gene cluster, the pattern of isotopic incorporation, and the pattern of dynaplanin production in our heterologous expression experiments, we considered a possible pathway for dynaplanin production (Fig. 4). Our isotopic labeling experiments showed that the phenylacetyl group of dynaplanin originates from phenylalanine (see Fig. S2). We propose that the DynV gene product, which contains a β-decarboxylation domain, catalyzes the PLP-dependent decarboxylation of phenylalanine (molecule 6) to phenylethylamine (molecule 7). A KEGG pathway analysis of the S. coelicolor genome showed that it did not appear to have a phenylalanine decarboxylase that functions under normal physiological conditions. However, a BLAST search of the S. coelicolor genome showed that it contains one homologs of DynV (SCO3385), which may explain why *dynV* was not essential for dynaplanin production in our heterologous expression experiments. KEGG analysis also showed that the S. coelicolor genome encodes enzymes capable of converting phenylethylamine (molecule 7) to phenylacetyl coenzyme A (phenylacetyl-CoA; molecule 10) in three reactions potentially catalyzed by TynA/AofH, FeaB, and PaaK, respectively (see Table S7). We also note that TynA/AofH, FeaB, and PaaK are also present in the genome of *C. caeruleus* T094, where we propose they play an analogous role in dynaplanin production.

From phenylacetyl-CoA, we hypothesize that dynaplanin A is produced in two steps through the incorporation of arginine followed by addition of octanoic acid (Fig. 4). We propose that DynZ is responsible for the decarboxylation/condensation of arginine and phenylacetyl-CoA, forming a C-C bond between the α-carbon of arginine and the carbonyl carbon of phenylacetyl-CoA, yielding the intermediate 1-(4-amino-5-oxo-6-phenylhexyl)guanidine (molecule 11). Subsequently, DynY is proposed to catalyze a condensation reaction between the amine of the arginyl group with the carbonyl C of octanoyl-ACP/CoA to give dynaplanin A (molecule 1).

### Identification of 2-oxo acid dehydrogenase as the presumptive target of dynaplanin.

The structure of dynaplanin does not readily suggest a potential mechanism of action that leads to growth inhibition. However, during assay-guided purification using Arthrobacter globiformis ATCC 8010 as an indicator organism, we occasionally encountered colonies that grew within dynaplanin inhibition zones. Testing of *C. caeruleus* T094 solid culture plugs containing dynaplanins against lawns of these spontaneous *A. globiformis* mutants yielded significantly smaller inhibition zones compared to the wild type, indicating that these mutants exhibited partial resistance to dynaplanins (see Fig. S5A). To find which genes carried mutations in these *A. globiformis* strains, we sequenced the genome of wild type and eight partially resistant mutants and conducted a single nucleotide polymorphism (SNP) analysis. The genomes of all eight mutants had SNPs located in a single region of a single gene, annotated as the catalytic domain of the E2 subunit of a 2-oxo acid dehydrogenase (see Fig. S5B).

**FIG 5:**
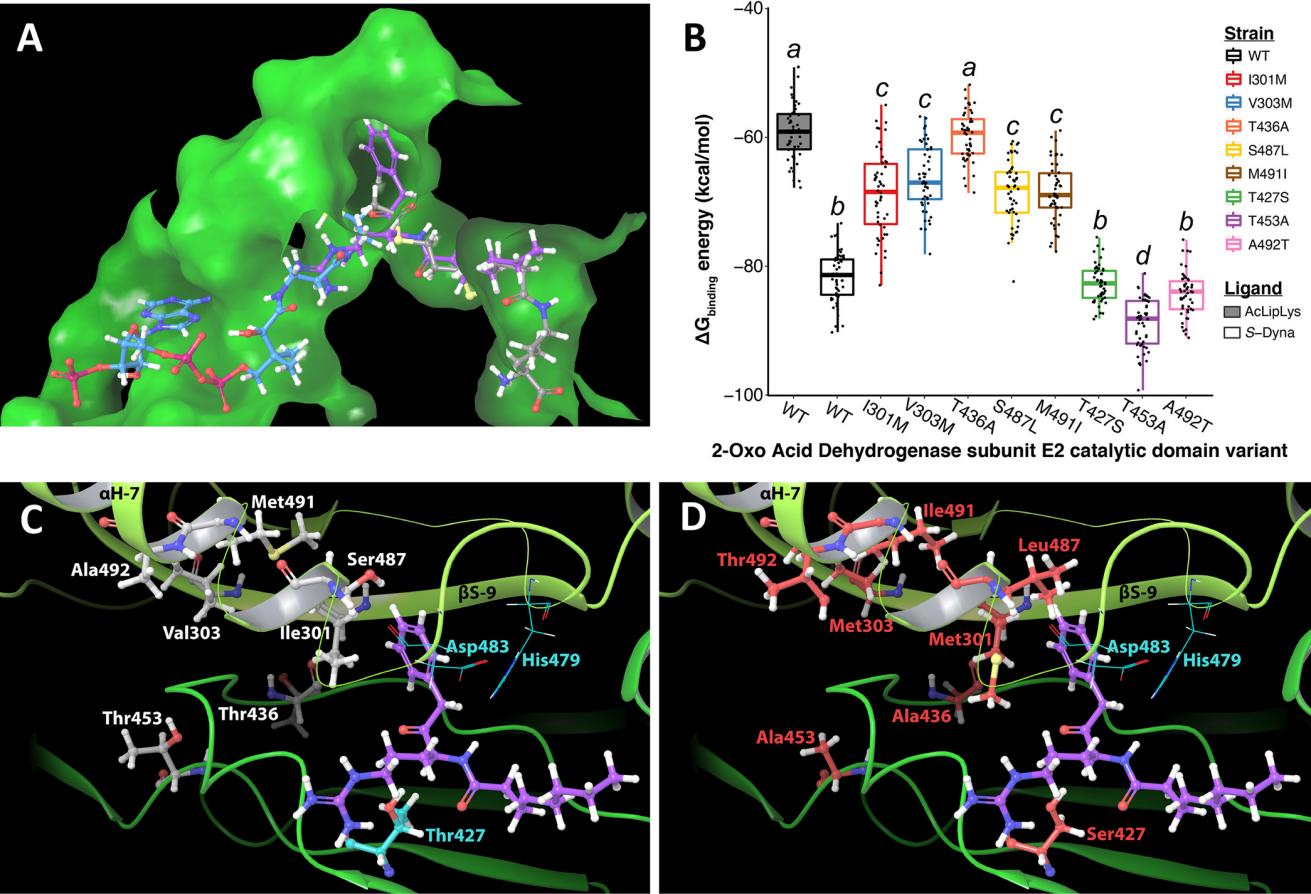
*S*-Dynaplanin A blocks native substrate binding in 2-oxo acid dehydrogenase subunit E2. (A) *S*-dynaplanin A docked in an *A. globiformis* 2-oxo acid dehydrogenase subunit E2 catalytic domain (E2-CD) model overlapping with both native substrates, coenzyme A (SH-CoA) and acetylated lipoyl-lysine (AcLipLys). Binding channel represented as green space-fill with 100% transparency over ligands and residues D483-G484 hidden for visual clarity. Ligands represented in ball-and-stick with carbons colored by ligand: *S*-dynaplanin A (purple), AcLipLys (gray), and SH-CoA (light blue). All other atom colors are defaults: hydrogen (white), oxygen (red), nitrogen (dark blue), phosphorus (pink), and sulfur (yellow). (B) Free energy of binding (Δ*G*_binding_) values (*n* = 50) calculated during a molecular dynamics simulation using the MM-GBSA algorithm. Ligands were bound in a trimer of *A. globiformis* E2-CD with wild-type residues or one amino acid substitution that lead to partial dynaplanin resistance (see Fig. S5 and Table S10 for more details). Whiskers extend 1.5 × IQR. ANOVA and Tukey’s *post hoc* tests were used to determine significance. Common letters represent samples that are not significantly different from each other but are significantly different from all other samples (*P* < 0.01). (C and D) Location and identity of wild-type residues (C, white carbon atoms) and resistant residues (D, red carbon atoms), including the catalytic triad (neon blue) in proximity to *S*-dynaplanin A.

The enzymatic mechanism of 2-oxo acid dehydrogenase complexes has been extensively studied at the structural level (48–57). The multiple available crystal structures for 2-oxo acid dehydrogenase complexes, and the conserved nature of these enzymes, enabled high-confidence modeling of the 2-oxo acid dehydrogenase complex whose gene was mutated in the partially resistant *A. globiformis* ATCC 8010 strains. When the eight amino acid substitutions were mapped onto this structural model, a clear pattern was apparent. All eight mutations led to residue substitutions within the E2 catalytic domain (Fig. 5C and D), with six of the eight substitutions (I301, V303, T436, S487, M491, and A492) occurring on or near alpha helix 7 and beta sheet 9, both of which are in proximity to the active site where chemical exchange between the acetyl-lipoyllysine and SH-CoA substrates occurs. This arrangement of residue substitutions suggests that the dynaplanins may bind in this region within the E2 catalytic domain.

To address the possibility that dynaplanins may bind in or near the active site of the 2-oxo acid dehydrogenase E2 catalytic domain, we took a computational modeling approach (see the “Extended Explanation” in the supplemental material for more details). The dynaplanin pose with the lowest docking score, indicating the most energetically favorable conformation, placed *S*-dynaplanin A in the substrate binding channel at the juncture where transfer of the acetyl moiety from the acetyl-lipoyllysine to the thiol on the CoA is catalyzed (Fig. 5A). Next, to get a more accurate estimate of the protein-ligand interactions, we performed molecular dynamics simulations with Desmond (Schrödinger, LLC) and then calculated free energy of binding for each ligand (acetyl-lipoyllysine, SH-CoA, and *S*-dynaplanin A). The native substrate acetyl-lipoyllysine exhibited a median Δ*G*_binding_ of −59.19 kcal/mol (Fig. 5B). *S*-Dynaplanin A showed a lower median Δ*G*_binding_ of −81.33 kcal/mol (Fig. 5B), indicating significantly more favorable binding in comparison to acetyl-lipoyllysine.

We next repeated these simulations with proteins carrying the amino acid substitutions observed in the partially resistant strains of *A. globiformis* (see Fig. S5). In five of the mutant proteins, we observed significantly increased Δ*G*_binding_ for *S*-dynaplanin A (I301M, V303M, T436A, S487L, and M491I) compared to the wild type (WT) (Fig. 5B). Notably, in one mutant enzyme, T436A, the Δ*G*_binding_ for *S*-dynaplanin A was statistically indistinguishable from that of acetyl-lipoyllysine (Fig. 5B). When we repeated these simulations with *R*-dynaplanin A, we found that its Δ*G*_binding_ was indistinguishable from acetyl-lipoyllysine in the WT protein and showed improved binding in seven of the eight mutant proteins (see Fig. S6D), which is inconsistent with those mutations causing a dynaplanin resistance phenotype. Amino acid interaction analysis using the Glide “score-in-place” algorithm (Schrödinger, LLC) indicated that all of the amino acid substitutions seen in the mutants led to changes in interaction energies and/or intermolecular distances between *S*-dynaplanin A and the surrounding residues, including the catalytic residues Asp483 and His479 (see Tables S8 and S9). Together, these results suggest that the *A. globiformis* strains (I301M, V303M, T436A, S487L, and M491I) are partially resistant to dynaplanins because *S*-dynaplanin A binds less favorably in the mutant enzymes.

In two mutant strains (T427S and A492T), *S*-dynaplanin A binding was not significantly different from WT, while one mutant (T453A) showed a significantly lower binding energy compared to WT (Fig. 5B). To understand why these *A. globiformis* strains are resistant to dynaplanins without worsening *S*-dynaplanin A binding, we calculated how the Δ*G*_binding_ for acetyl-lipoyllysine and SH-CoA changed in the mutant backgrounds compared to the WT protein. All of the amino acid substitutions (except S487L) significantly decreased Δ*G*_binding_ for acetyl-lipoyllysine (see Fig. S6B and Table S10), suggesting that the native substrate may bind better in these mutant backgrounds than in WT. With acetyl-lipoyllysine bound, the Δ*G*_binding_ for SH-CoA also decreased significantly in T427S and A492T enzymes compared to WT (see Fig. S6F and Table S10). Taken together, our simulation results suggest that the additive effects of improved acetyl-lipoyllysine and SH-CoA binding may explain why T427S and A492T mutants are resistant to dynaplanins, even though *S*-dynaplanin A Δ*G*_binding_ is not significantly different from the WT in these backgrounds.

## DISCUSSION

Microbial genome sequencing efforts of the past 2 decades have revealed an abundance of natural product biosynthetic gene clusters that have yet to be associated with a metabolite, presumably because these gene clusters are poorly expressed under typical laboratory conditions. Accessing this latent reservoir of chemical diversity is therefore of high interest, and multiple strategies have been developed to tap into this resource. These strategies include genome mining approaches that rely on a wealth of genomic information (6, 58, 59), metagenome-guided biosynthesis (60, 61), chemical profiling of microbial interactions (reviewed in references 28 and 62), enriching for likely producers based on intrinsic antimicrobial resistance (63), and harnessing compound libraries as elicitors of silent gene cluster activation (HiTES) (23, 24, 64). Each of these strategies has their own unique advantages (reviewed in references 2, 24, and 65). However, most of them also involve labor-intensive genetic manipulation, high-throughput culturing/chemical analysis, or a combination of both, to increase the likelihood of discovering novel natural products. In the present study, we pursued a simplified strategy that harnessed multiple aspects of actinomycete ecology to maximize the probability of finding novel chemical scaffolds. This strategy enabled the discovery of the dynaplanins, which appear to inhibit microbial growth through an unusual mechanism of action, and whose gene cluster was missed by typical genome mining tools for natural product biosynthetic gene cluster detection.

We started by selecting a small number of strains (eight), with an emphasis placed on maximizing phylogenetic diversity across rare actinomycetes, in an effort to increase the likelihood that the strains would contain gene clusters that differ from each other and those commonly found in *Streptomyces* species (1, 66, 67). Next, we grew the strains in binary interactions. A growing body of research has shown that natural product biosynthesis can be stimulated through interspecies interactions, which are mediated by competition for resources, exchange of chemical cues, or other mechanisms (26–28). We next overlaid the interactions directly with an indicator strain (45), *Amycolatopsis* sp. AA4, that has high intrinsic resistance to a wide array of at least 15 antimicrobials (42). In doing so, we aimed to exploit this high level of natural resistance to avoid detecting commonly produced antimicrobials, thus enhancing the likelihood that any positive hits would be of a novel class. At each step, from initial strain selection to growth conditions, to detection, our strategy was designed to increase the probability of uncovering unusual microbial natural products. This strategy has several advantages: it is technically simple to implement, requires no genetic manipulation, uses only 16S typing to enable diverse strains to be chosen, and in this case, the throughput required to detect a novel compound was low (28 interactions). One caveat to this strategy is that with no *a priori* information about the genomic content of the input strains, the ability of the researcher to bias the discovery output toward a desired chemical class is limited. This could be circumvented by including input strains for which genome sequences are available, similar to the other genome-guided strategies noted above.

The antimicrobial compounds detected in our screen, the dynaplanins, constitute a unique bioactive scaffold consisting of a central arginine residue modified with a phenylacetyl moiety and an acyl chain. Despite being relatively simple in terms of chemical structure, identifying the gene cluster responsible for dynaplanin production was complicated by the fact that it is not produced by enzymes found in typical natural product pathways (e.g., nonribosomal peptide synthetases, polyketide synthases, or terpene synthases). Our stable isotope labeling experiments/sequence analyses indicate that the phenylacetyl group of dynaplanin originates from phenylalanine and likely proceeds through a phenylacetyl-CoA intermediate. We propose that the phenylacetyl moiety is transferred to the α-carbon of the arginine via a Claisen-type decarboxylation/condensation reaction catalyzed by the *dynZ* gene product. DynZ belongs to a superfamily of PLP-dependent *C*-acetyltransferases, including 5-aminolevulinate synthase (ALAS), and 8-amino-7-oxononanoate synthase (AONS), and searching the genome of the producing organism for such *C*-acetyltransferases ultimately led to identification of the *dyn* gene cluster. Notably, *dynZ* and *dynY* were the only two genes essential for dynaplanin production in a heterologous host.

A few other natural product biosynthetic pathways, including those of ECO-02301 (68), asukamycins (69), colabomycins (70), annimycins (71), and ketomemicins (47), are thought to employ stand-alone PLP-dependent *C*-acetyltransferases (also reviewed in reference 46). This study adds DynZ to the repertoire of such enzymes and provides a potentially new avenue for introducing the phenylacetyl moiety onto synthetic peptides through an atypical α-carbon linkage. Additional biosynthetic studies will be required to assess DynZ substrate specificity and potential utility in this regard, including the absolute configuration of C-2, which was not determined here. While the nonpeptide bond linkages seen in the arphamenines and ketomemicins (i.e., via carbonylmethylene) differ from that seen in the dynaplanins, these three molecule families are likely united by their use of PLP-dependent *C*-acetyltransferases (47, 72) to incorporate phenyl amino acid derivatives into pseudopeptides containing arginine. This observation prompts us to speculate that other similarly unusual arginyl pseudopeptides may await discovery and that exploring genomes for *dynZ*-type homologs may be a route to their discovery.

Two lines of evidence, including sequencing of partially resistant mutants and *in silico* enzyme docking studies, indicate that the likely target of the dynaplanins is the catalytic domain of the E2 subunit of a 2-oxo acid dehydrogenase complex. 2-Oxo acid dehydrogenases are metabolic enzymes that catalyze the decarboxylation of 2-oxo acids, producing a corresponding acyl-CoA intermediate. Most organisms contain three types of 2-oxo acid dehydrogenase complexes, namely, the pyruvate dehydrogenase complex (PDC), the 2-oxoglutarate dehydrogenase complex (OGDC), and the branched-chain 2-oxo acid dehydrogenase complex (BCOADC). The high sequence similarity across these enzymes makes it challenging, if not impossible, to differentiate between these three possibilities based on homology analyses. During the 2-oxo acid dehydrogenase reaction cycle, the E1 domain catalyzes the decarboxylation and transfer of the substrate to a lipoamide cofactor that is covalently bound to a lysine in the lipoyl domain of the E2 subunit (reviewed in reference 73). This “swinging arm” domain then introduces the substrate-bearing lipoamide cofactor into the active site channel of the E2 subunit, where the substrate is transferred to a CoA molecule (reviewed in reference 74).

Six of the eight mutations we observed in partially resistant strains were in locations that likely lead to shifts in the structure of the substrate binding channel around the E2 active site, suggesting that dynaplanins might bind within this region. This hypothesis is appealing since dynaplanin binding in this region could occlude the normal insertion of either the substrate-carrying lipoyllysine or the CoA into the E2 active site. In line with this hypothesis, our docking studies indicated that the most likely pose for *S*-dynaplanin A is one in which the arginyl moiety of dynaplanin A occludes the CoA, while the octanoic acid moiety occludes the substrate-carrying lipoyllysine. We note that in our molecular dynamic simulations, five of the mutations led to significantly decreased binding of *S*-dynaplanin A, and seven of the mutations led to enhanced binding of either/or both acetyl-lipoyllysine or SH-CoA. Thus, these simulations demonstrate the plausibility of this potential mechanism of action and set the stage for future studies to directly test these hypotheses.

Interest in 2-oxo acid dehydrogenase complexes as potential targets for antimicrobials extends back at least 4 decades (75, 76), and these enzymes are also of keen interest for inhibition given their role in oncogenesis (77, 78) and neurodegenerative disease (79, 80). However, only a few small molecule inhibitors of these enzymes have been identified, with all of them affecting the E1 subunit of the complex (75, 76, 81–87). The only natural product among these is dehydrophos, which appears to require cleavage of its peptide bonds to yield a toxic pyruvate mimic, methyl acetylphosphonate, which subsequently inhibits multiple pyruvate-utilizing enzymes, including the pyruvate dehydrogenase complex (83, 88). Thus, to our knowledge, dynaplanins are the first identified natural products that appear to inhibit the E2 subunit of a 2-oxo acid dehydrogenase. Our findings also indicate that dynaplanins have a relatively narrow spectrum of inhibition, being active primarily against a subset of other actinomycetes at relatively high concentrations. The determinants of this narrow spectrum, be it from nonspecific mechanisms such as drug efflux or specific features of the 2-oxo acid dehydrogenase complex of sensitive actinomycetes, is an area for further exploration. Analyzing the primary structure of 2-oxoacid dehydrogenases of organisms predicted to produce dynaplanins may offer further clues in this regard.

What role might dynaplanins play in natural settings? A wealth of studies suggest that soil microbial communities are arenas where competition is fierce and resistance to antimicrobials is common (36, 39, 89–91). In such an environment, microbes are likely under pressure to chemically innovate novel ways of inhibiting their competitors. Dynaplanins, which appear to directly inhibit primary metabolism, may afford their producers an advantage in this landscape, especially where exploitative competition is strong. An alternative hypothesis is that dynaplanins may serve as a mechanism used by the producing organism to modulate flux through their own primary metabolic pathways. In either case, this work highlights the importance of incorporating ecological factors into natural products discovery efforts, and illustrates that doing so, even at a small scale, can lead to the discovery of molecules with unusual modes of antimicrobial activity.

## MATERIALS AND METHODS

### Strains and culturing conditions.

Strains used in this study are listed in Table S1. All actinomycetes were grown on ISP2 agar medium (10 g of malt extract, 4 g of glucose, 4 g of yeast extract, 15 g of agar, 1 L of Milli-Q H_2_O) and nonactinomycetes were grown on Luria-Bertani (LB) agar medium (10 g of tryptone, 5 g of yeast extract, 5 g of NaCl, 15 g of agar, 1 L of Milli-Q H_2_O), unless otherwise indicated. Plates were sealed with parafilm to prevent desiccation and incubated at 30°C. Escherichia coli strains were grown on LB medium with appropriate antibiotics, where relevant, followed by incubation at 37°C. For dynaplanin activity tests, Actinomyces israelii strain 277 was grown on brain heart infusion agar (BD Bacto) and incubated at 37°C under anaerobic conditions in a BD GasPak EZ container system. Enterococcus faecalis (VRE) was grown on tryptic soy agar (TSA; BD Bacto) and incubated at 30°C. For solo patch experiments and stable isotope labeling, Couchioplanes caeruleus strains were grown on a modified ISP4 medium [called ISP4g+MOPS: 10 g of glucose, 1 g of K_2_HPO_4_, 1 g of MgSO_4_, 1 g of NaCl, 2 g of (NH_4_)_2_SO_4_, 2 g of CaCO_3_, 1 mg of FeSO_4_·7H_2_O, 1 mg of MnCl_2_·7H_2_O, 1 mg of ZnSO_4_·7H_2_O, 20 g of agar, 100 mL of 10× MOPS, filled to 1 L of Milli-Q H_2_O]. *Couchioplanes* sp. were initially isolated from soil samples using the rehydration/centrifugation method, as described in Hop et al. (32).

Initial screening of binary interactions was performed in 12-well plates with 0.75 mL of ISP2 agar per well. One microliter of frozen spore stock solution was spotted in the center of the well (alone) or 5 mm apart from center to center for interacting strains. Strains were tested in duplicate and incubated at 30°C for 5 days. A soft agar overlay (0.75% agar + 0.5× ISP2) of the indicator strain (*Amycolatopsis* sp. AA4) was applied over the interacting species, allowed to cool, and incubated at 30°C overnight to assess zones of clearance, as originally described by Ueda et al. (45). For followup interaction experiments, interactions and solo patches were grown in 60 × 15-mm petri dishes with 4 mL of ISP2 or ISP4g+MOPS agar, depending on the species. One microliter of each species’ freezer stock was spotted in the center of the well (alone) or 1 cm apart from center to center of the interacting strain. Plates were sealed with parafilm and incubated at 30°C for 5 days. A soft agar overlay (0.75% agar + 0.5× ISP2) of the indicator strain (*Amycolatopsis* sp. AA4 or Arthrobacter globiformis) was applied over the interacting species, allowed to cool, and incubated at 30°C overnight to assess zones of clearance.

For plug assays, *C. caeruleus* T094 was spread plated to high cell density (1 × 10^5^ CFU/mL) on either ISP2 or ISP4g+MOPS agar. An agar plug was punched out using a straw with a plastic plunger. The plug was placed on top of agar which previously had a lawn of indicator strain spread across it. Plates were incubated agar side down for 1 day at 30°C.

For quantifying the resistance of *A. globiformis* mutants, single colonies of *A. globiformis* spontaneous dynaplanin-resistant mutants (K, M, O, P, Q, R, Y, and BB) and wild type used to inoculate overnight liquid cultures of ISP2, which was incubated at 30°C with shaking. Next, 20-μL portions of these cultures were spread evenly with glass beads on 4-mL ISP2 plates (15 × 60 mm) in triplicate. One agar plug from a 6-day T094 culture was placed in the center of each plate. Plates were sealed with parafilm and incubated at 30°C for 1 day with the agar side down. Diameters of zones (in mm) were measured.

For stable isotope labeling experiments, *C. caeruleus* T094 was spread plated to high cell density (1 × 10^5^ CFU/mL) on ISP4g+MOPS agar supplemented with either l-[^13^C_6_]-arginine (5.2 mM in H_2_O), l-[^13^C_9_^15^N]-phenylalanine (4.2 mM in H_2_O + a few drops of NaOH), or 1,2,3,4-^13^C_4_]-octanoic acid (100 μM in ethanol). Plates were incubated at 30°C for 3 days (L-Arg and L-Phe) or 4 days (Oct). Plates were extracted in ethyl acetate as described below and analyzed via LC-MS/MS.

### Dynaplanin extractions, sample preparation, and purification.

Agar plates were extracted with ethyl acetate (1:1 [vol/vol]) for 1 to 2 h after being sonicated for 10 min. The extract was then poured off, and then a fresh batch of ethyl acetate was added (1:1 [vol/vol]) and stored overnight in a chemical fume hood. Both batches were combined and dried in a rotary evaporator or SpeedVac SPD-1010-115 (Thermo Fisher). Samples were resuspended in 100% methanol to approximately 1 mg/mL and sonicated for 10 min. Samples were transferred to 1.5-mL microcentrifuge tubes and centrifuged at 15,000 rpm for 2 min to ensure that no particulates were added to LC-MS vials. Samples were combined 1:1 with an internal standard, reserpine (20 μg/mL), in LC-MS vial glass inserts.

For purification, *C. caeruleus* T094 was spread at high cell density (1 × 10^5^ CFU/mL) on 150 mm × 15-mm plates containing 40 mL of ISP2 agar medium. Plates were incubated at 30°C for 4 days. Plates were extracted as described above. Crude extract was subject to solid-phase extraction (SPE) for partial purification using a C-18 Sep-Pak Vac 35 cc, 10-g column (Waters). Extract was loaded on the column in 25% methanol (MeOH) and eluted in increasing concentrations: 25, 50, 75, and 100% MeOH. The 100% MeOH fraction was injected on LC-MS/MS to optimize peak separation prior to semipreparative LC using a C-18 column (250 mm × 4.6 mm, 5.0 μm, 120 Å; Thermo Scientific Acclaim RSLC) and a 1.0-mL/min flow. Dynaplanins did not exhibit a strong UV absorbance profile, and bioassay-guided fractionation was paired with LC-MS/MS. Semipreparative LC was run on an ultra-high pressure liquid chromatography (UHPLC) system (Dionex Ultimate 3000, Thermo Fisher) with a C_18_ column (250 mm × 10 mm, 5.0 μm, 120 Å; Thermo Scientific Acclaim RSLC). The SPE fraction containing dynaplanins was dried and resuspended in 50% MeOH after 10 min of sonication. LC was run at 4.7-mL/min flow with water (solvent A) and acetonitrile (solvent B) under the following gradient: 30% B for 0.0 to 2.0 min, 30 to 55% B for 2.0 to 2.25 min, 55 to 65% B for 2.25 to 12.25 min, 65 to 99% B for 12.25 to 15.0 min, 99% for 15.0 to 21.0 min, 99 to 30% for 21.0 to 25.0 min, and 30% from 25.0 to 30.0 min. Multiple injections of 150 μL were run, and fractions were pooled. Fractions were collected every minute from 3.0 to 27.0 min and dried in a SpeedVac SPD-1010-115 (Thermo Fisher). Fraction 10 (12.0 to 13.0 min) was a pure molecule of [M+H]^+^ = 375.2746. This molecule was named dynaplanin A (molecule 1).

### LC-MS/MS analysis.

Samples were analyzed using a UHPLC system (Dionex Ultimate 3000; Thermo Fisher) coupled to a high-resolution mass spectrometer (HRMS, Thermo Q-Exactive Quadrupole-Orbitrap; Thermo Fisher) using a heated electrospray ionization source. Samples were injected (5.0 to 10.0 μL) on a C_18_ column (50 mm × 2.1 mm, 2.2 μm, 120 Å; Thermo Scientific Acclaim RSLC) with a gradient of 0.1% formic acid in water (solvent A) and 0.1% formic acid in acetonitrile (solvent B) at a 0.4-mL/min flow rate. The gradient was as follows: 30% B for 0.0 to 1.5 min, 30 to 99% B for 1.5 to 12.0 min, 99% for 12.0 to 15.0 min, 99 to 30% for 15.0 to 15.5 min, and 30% from 15.5 to 17.0 min. The column oven was set to 35°C. Analyses were run in centroid mode. Full MS1 scanning was performed in positive mode, with a resolution of 70,000 full width at half-maximum (FWHM), the automatic gain control (AGC) target was set at 1 × 10^6^ ions with a maximum ion injection time (IT) of 100 ms, mass range from *m/z* 100 to 650. For LC-MS/MS analysis, the MS/MS data were acquired using the data-dependent analysis mode, in which the five most intense ions were sent for fragmentation (Top5 method), excluding repetitive ions for 5 s (dynamic exclusion), at a resolution of 17,500 FWHM, with an AGC target of 1 × 10^5^ ions, and a maximum IT of 50 ms, using an isolation window of 3 *m/z* and normalized collision energies of 20, 30, and 40. The cone spray voltage was 3.5 kV. Theoretical [M+H]^+^ of dynaplanins A to E (molecules 1 to 5) and unnamed analogs were calculated as described elsewhere (https://warwick.ac.uk/fac/sci/chemistry/research/barrow/barrowgroup/calculators/mass_errors), an approach which was also used to calculate mass error. Extracted ion chromatograms (EICs) and tandem mass spectra (MS2) results were loaded into R, processed with the data.table package, and plotted with the ggplot2 package. EICs of dynaplanin A ([M+H]^+^ range, 375.2746 ± 5 ppm) were normalized to the maximum dynaplanin A peak per sample.

### Nuclear magnetic resonance analysis.

Dynaplanin A (molecule 1) was dissolved into 140 μL of DMSO-*d*_6_ (Cambridge Isotope Laboratories, Inc.) and then transferred to a 2.5-mm NMR tube. The ^1^H, ^13^C, ^1^H–^1^H COSY, ^1^H–^13^C HSQC, and ^1^H–^13^C HMBC NMR spectra were acquired, respectively, on a Bruker Avance 900 NMR spectrometer (900 MHz for ^1^H and 225 MHz for ^13^C) equipped with a cryoprobe. Data were collected and reported as follows: chemical shift, integration multiplicity (s, singlet; d, doublet; t, triplet; m, multiplet) and coupling constant. Chemical shifts were reported using the DMSO-*d*_6_ resonance as the internal standard for ^1^H NMR DMSO-*d*_6_: δ = 2.50 ppm and ^13^C NMR DMSO-*d*_6_: δ = 39.5 ppm.

### Dynaplanin A MIC.

MIC of dynaplanin A (molecule 1) was measured against *A. globiformis*, *Amycolatopsis* sp. AA4, E. coli, and B. subtilis using a standard broth microdilution method (92). Briefly, dynaplanin A (in 6% dimethyl sulfoxide) was diluted 2-fold in a range from 200 to 0.20 μg/mL. All strains were tested in triplicate in Mueller-Hinton broth, grown at 30°C shaking at 1,200 rpm in a 96-well heated plate shaker (Southwest Science). The *A*_600_ of each well was measured in a Tecan Spark plate reader after 18 h of incubation. MICs were determined from the wells with the lowest concentration of dynaplanin A that resulted in no growth of the species compared to a dynaplanin A blank control.

### *C. caeruleus* T094 genome sequencing, assembly, annotation, and analysis.

Genomic DNA was extracted from *C. caeruleus* T094 that was grown on ISP2 for 7 days. DNA quality was confirmed using gel electrophoresis, a NanoDrop One UV-Vis spectrophotometer, and a Qubit fluorometer (Invitrogen Qubit DNA-HS assay kit, Q32851). Genomic DNA was run through the UCB QB3 PacBio Sequel II sequencing pipeline. Raw reads were assembled *de novo* using the Flye assembler (version 2.7.1) (93) into one scaffold with an average 993× coverage. The draft genome was annotated using the NCBI Prokaryotic Genome Annotation Pipeline (PGAP) (94).

For natural product biosynthetic gene cluster prediction, the *C. caeruleus* T094 and DSM43634 genomes were submitted to antiSMASH version 6.0.1 (95) using relaxed conditions. Default “extra features” selected included KnownClusterBlast, ActiveSiteFinder, SubClusterBlast, and RREFinder. For global metabolic gene analysis, the *C. caeruleus* T094 genome was submitted to BlastKOALA (96) for K-number assignment and visualization of metabolic pathways in KEGG. For comparative analysis of the dynaplanin biosynthetic gene cluster, sequences were manually uploaded to the Gene Graphics (97) web application for comparison of gene organization.

### Genetic manipulation of the *dynVWXYZ* gene cluster.

The *dynVWXYZ* gene cluster was amplified from *C. caeruleus* T094 genomic DNA using primers in Table S6. The full cluster (~3.7 kb) was inserted into the *lacZ* gene of pSET152 by Gibson assembly (98). Plasmids were electroporated into E. coli TOP10 and plated on LB+IPTG+X-Gal+apramycin to conduct blue-white screening and plasmid selection. White colonies (lack of LacZ activity) were picked, and plasmids recovered by a QIAprep spin miniprep kit (Qiagen). Recovered plasmids were sequenced at the UC Berkeley DNA Sequencing Facility to confirm no mutations in the *dynVWXYZ* cluster. Plasmids were electroporated into E. coli ET12567/pUZ8002, which contains the conjugation transfer genes, and selected on LB medium with appropriate antibiotics.

Single deletions of *dynVWXYZ* genes from plasmid pDM2 were constructed with primers in Table S6. Two fragments were amplified on either side of the gene to be deleted and were Gibson assembled. The *dynV* and *dynW* gene deletions left only start and stop codons. For the *dynX*, *dynY*, and *dynZ* gene deletions, the maximum amount of DNA was deleted that did not affect overlapping open reading frames. Plasmids were prepped for conjugation using the workflow described above. Plasmid constructs were conjugated into S. coelicolor M145 using E. coli ET12567/pUZ8002. Exconjugants were grown at 30°C on mannitol soy agar supplemented with MgCl_2_ (10 mM) and CaCl_2_ (60 mM) for efficient conjugation frequency. Genomic DNA was isolated to confirm plasmid integration by PCR, and genes were sequence verified.

### LC-MS/MS confirmation of dynaplanin A production in *S. coelicolor* heterologous expression hosts.

S. coelicolor heterologous expression strains and *C. caeruleus* T094 were grown on ISP4g+MOPS agar plates with no supplements or supplemented with all three isotope labeled substrates (400 μM l-[^13^C_6_]-arginine, 400 μM l-[^13^C_9_^15^N]-phenylalanine, and 100 μM [1,2,3,4-^13^C_4_]-octanoic acid) for 5 days at 30°C. Labeled isotopes were used to validate the presence of dynaplanins in addition to MS2 spectra. S. coelicolor with the Δ*dynX* plasmid were unable to grow on ISP4g+MOPS plates. Thus, S. coelicolor with *dynVWXYZ*, with the Δ*dynX* plasmid, or with the empty vector were grown on ISP2 agar plates for 5 days at 30°C. Strains were plated in triplicate. Next, ten agar plugs from each plate were extracted for 1 h in ethyl acetate, dried in a SpeedVac SPD1010-115 (Thermo), and resuspended in 100% MeOH. Samples were analyzed via LC-MS/MS as described above. EICs were loaded into R, processed with the data.table package, and plotted with the ggplot2 package. The EICs of dynaplanin A ([M+H]^+^ range, 375.2746 ± 5 ppm) were normalized to the maximum dynaplanin A peak in T094. Dynaplanin A (molecule 1) was verified by MS2 in all S. coelicolor heterologous hosts that produced it.

### Genomic DNA preparation for Illumina sequencing of *A. globiformis* mutants and SNP analysis.

Genomic DNAs of Arthrobacter globiformis wild type and spontaneous resistance mutants (K, M, O, P, Q, R, Y, and BB) were extracted by using a Qiagen DNeasy blood and tissue kit with optimized cell lysis for Gram-positive bacteria. DNA quality was confirmed by using gel electrophoresis, a NanoDrop One UV-Vis spectrophotometer, and a Qubit fluorometer (Invitrogen Qubit DNA-HS assay kit, catalog no. Q32851). Library preparation and sequencing were performed by UCB QB3 with Illumina NovaSeq 150-bp, paired-end reads. SNPs and indels were identified in mutants and the wild type by aligning raw, paired-end reads to the *A. globiformis* reference genome NBRC12137 (NCBI accession NZ_BAEG01000085) individually using Snippy (version 4.5.0; https://github.com/tseemann/snippy) with standard parameters. Snippy analyses were run on the Galaxy (99) computing cluster. SNPs/indels with 150 raw reads or fewer were excluded from the analysis.

### Computation protein modeling of the *A. globiformis* 2-oxo acid dehydrogenase subunit E2 catalytic domain and substrate binding simulations. (i) Preprocessing of proteins.

The *A. globiformis* 2-oxo acid dehydrogenase E2 protein (gene locus RS19070) was run through Phyre2 using intensive mode. The resulting three-dimensional (3D) structure was imported into Maestro, run remotely through the Molecular Graphics and Computation Facility (MGCF) at Berkeley. Residues M1-D259 were removed from *A. globiformis* E2 since most available crystal structures include only the catalytic domain (CD) plus several unstructured residues preceding it (48–50, 52–55, 57). The Corynebacterium glutamicum E2pCD (PDB 6ZZK) (48) with both CoA and dihydrolipoamide bound was used as a reference. Standard protein preprocessing steps were run, including filling in missing loops and side chains with Prime, hydrogen bond optimization (pH 7.0 with PROPKA), water molecules removed (beyond 3.0 Å and with fewer than three hydrogen bonds to nonwater molecules), and root mean square deviation (RMSD) minimization with OPSL4 set to 0.3 Å. This was repeated for all three monomers of the C. glutamicum E2pCD trimer. This trimer was used to build a homology model of the *A. globiformis* E2-CD. Then, the protein preprocessing steps described above were repeated for the *A. globiformis* E2-CD monomer, which was duplicated twice and aligned to each monomer of the C. glutamicum E2pCD trimer (Prime, release 2021-4; Schrödinger, LLC, New York, NY).

### (ii) Ligand preparation and receptor grid generation.

Ligands were prepared using the following LigPrep parameters: pH 7.0 ± 2.0 using Epik, OPLS4 force field, desalt and generate tautomers (Schrödinger release 2021-4; LigPrep). Chirality was locked based on the 3D structure for all ligands. Using the dihydrolipoamide reference position from 6ZZK, a receptor grid was generated at the interface of monomers 1 and 3 using default parameters. All rotatable bonds within the 5.0 × 5.0 × 5.0 Å^3^ grid space were allowable. In *A. globiformis*, potential hydrogen bond donors and receptors included: Thr285, His479, and Asn442, based on the Azotobacter vinelandii active site (49, 54). In C. glutamicum, potential hydrogen bond donors and receptors included Thr451, His645, and Val604, based on proximity to the residues in *A. globiformis*.

### (iii) Ligand docking.

Acetyl-lipoyllysine (AcLipLys) and *S-*dynaplanin A were docked with Glide (standard precision) in the *A. globiformis* E2CD using the grid above (Schrödinger release 2021-4; Glide). Acetyl-lipoyllysine was used since typical 2-oxo acid dehydrogenases must minimally accommodate acetyl-lipoyllysine, i.e., the PDC uses acetyl-lipoyllysine directly, while OGDC and BCOADC each accommodate substrates that are bulkier than acetyl-lipoyllysine. For AcLipLys, parameters were default except vdW scale = 0.7, at least one hydrogen bond to any residue defined in the grid, and 0.0 kcal/mol maximum threshold for output poses. The modified parameters that generated *S-*dynaplanin A poses were as follows: vdW scale = 0.8 and zero hydrogen bonds necessary. AcLipLys docked in C. glutamicum 6ZZK with the same parameters above except vdW scale = 0.75 and no hydrogen bond constraints were necessary. The X-ray crystal reference positions for dihydrolipoamide and CoA were used in both C. glutamicum 6ZZK and *A. globiformis* E2-CD. Neither ligand was docked; however, the Glide score-in-place algorithm was used for dihydrolipoamide in both proteins.

### (iv) Molecular dynamics simulations and calculating the free energy of binding (Δ*G*_binding_).

In all trimers, CoA and dihydrolipoamide in the 6ZZK reference position were placed in their respective binding sites. Only ligands docked in place of dihydrolipoamide in the interface of monomers 1 and 3 were varied. Trimers with ligands bound were solvated using the SPC function, the solvent box volume was minimized, and NaCl salt was added to 0.15 M. Molecular dynamics simulations were run with Desmond (Schrödinger release 2021-4; Desmond Molecular Dynamics System, D. E. Shaw Research, New York, NY). Maestro-Desmond Interoperability Tools, Schrödinger, New York, NY) at 303.15 K (30°C) for 10 ns, with an interaction analysis run when the simulation finished. All docked poses above were run using molecular dynamics in the wild-type protein (*A. globiformis* and C. glutamicum) and single amino acid substitution mutants (*A. globiformis*). Molecular dynamics trajectories were centered on the ligand of interest using the trj_center.py command. Then Δ*G*_binding_ was calculated with MM-GBSA every 0.1 ns from 5.0 to 10.0 ns (*n* = 50) using the thermal_mmgbsa.py script.

### (v) Amino acid interaction analyses.

The median Δ*G*_binding_ value for *S-*dynaplanin in each molecular dynamic simulation (wild type and mutants) was used to measure amino acid interactions with the ligand. The median “frame” from the simulation trajectory was extracted, and water molecules >3.0 Å away from binding sites and nonwater molecules were removed. CoA was removed from the binding site to create a secondary grid around *S-*dynaplanin A using default parameters (see above for details). The Glide score-in-place algorithm was used to calculate amino acid interactions that were within 12.0 Å of *S-*dynaplanin A. During simulations, ligands (i.e., *S-*dynaplanin A) move within the binding site, and not all amino acids are within 12.0 Å in all simulation frames. A subset of residues that are common to all frames were compared by subtracting the wild-type frame values from each mutant frame values. Interaction energies and distances from *S*-dynaplanin A are presented as a heat-map in Tables S8 and S9.

### (vi) Image processing.

All protein images were taken directly in Maestro. Amino acid labels were added using Adobe Photoshop.

### Statistical analyses and box-and-whisker plot generation.

One-way analysis of variance (ANOVA) and *post hoc* Tukey HSD tests were performed on Astatsa.com to determine significance. In all graphs, shared letters represent samples that are not significantly different from one another but are significantly different from all others (*P* < 0.01 unless otherwise indicated). MM-GBSA Δ*G*_binding_ results for each ligand were loaded into R, processed with the data.table package, and plotted with the ggplot2 package. The geom_boxplot function was used to calculate interquartile ranges (IQR) and generate box-and-whisker plots with default settings. Whiskers extend 1.5 × IQR.

### Data availability.

The *C. caeruleus* T094 genome is available at the NCBI under accession number CP092183. Supplemental material is accessible via Figshare at https://doi.org/10.6084/m9.figshare.19252106.v1.
